# Pulmonary Tuberculosis and SARS, China

**DOI:** 10.3201/eid1204.050264

**Published:** 2006-04

**Authors:** Wei Liu, Arnaud Fontanet, Pan-He Zhang, Lin Zhan, Zhong-Tao Xin, Fang Tang, Laurence Baril, Wu-Chun Cao

**Affiliations:** * Beijing Institute of Microbiology and Epidemiology, Beijing, People's Republic of China,; † Institut Pasteur, Paris, France;; ‡ Beijing Institute of Basic Medical Sciences, Beijing, People's Republic of China

**Keywords:** SARS, People’s Republic of China, pulmonary tuberculosis, letter

**To the Editor:** As part of a cohort study of 83 patients with severe acute respiratory syndrome (SARS) in Beijing, China, we conducted a follow-up study of all the patients by routine medical examination. During the process, 3 patients with chest radiographs consistent with active disease were identified as having pulmonary tuberculosis (TB). Here we describe the 1-year clinical outcome and immune response in these patients.

Demographic details and coexisting conditions are shown in the Table. Patient 1 was a healthcare worker who became infected with SARS-associated coronavirus (CoV) while on duty with SARS patients. After he was transferred to a hospital dedicated to SARS management, pulmonary TB was diagnosed (positive acid-fast bacilli smear on sputum samples). Patients 2 and 3 were known to have cases of pulmonary TB and became infected with SARS-CoV after contact with other patients hospitalized for SARS. These 2 patients were sputum smear–negative for acid-fast bacilli, and diagnosis was made on the basis of previous exposure to TB, relevant symptoms of typical pulmonary TB, chest radiographs consistent with active disease, a positive tuberculin skin test result, and the finding of cavity regression on chest radiographs after anti-TB treatment was initiated. No cultures were obtained for isolation and comparison of *Mycobacterium tuberculosis* strains (*1*). All 3 patients had confirmed SARS based on amplification of SARS-CoV RNA by reverse transcriptase–polymerase chain reaction (RT-PCR) from sputum and stool specimens (*2*). Patients 2 and 3 recovered without complications; patient 1 had the most severe disease and required mechanical ventilation in an intensive care unit before recovering.

**Table:** Demographic and clinical information on severe acute respiratory syndrome (SARS) patients with pulmonary tuberculosis (TB), Beijing, China, 2003

Demographic/clinical characteristic	Patient 1	Patient 2	Patient 3
Sex	M	M	M
Age (y)	48	18	20
Other coexisting conditions	*Pseudomonas aeruginosa* infection	None	None
Date of SARS onset	Apr 5, 2003	Apr 7, 2003	Apr 4, 2003
Date of TB diagnosis	Jun 12, 2003	Jan 24, 2003	Mar 5, 2003
Date of hospitalization or transfer to SARS ward	Apr 7, 2003	Apr 7, 2003	Apr 5, 2003
Leukocyte count at admission, /μL	12,500	6,800	2,500
Total steroid dose used, mg	25,280	2,600	3,600
Intensive care unit admission	Yes	No	No
CD4/CD8 cell ratio 6 mo after disease	0.63	1.56	1.23
Absolute CD4 cell count 6 mo after disease, /μL	368	431	306
Absolute lymphocyte count 6 mo after disease, /μL	2,098	1,115	1,666

Both cellular and humoral immunity were evaluated during the follow-up of these patients. T-lymphocyte subsets were measured 6 months after disease onset by flow cytometry using fluorescein isothiocyanate–labeled specific monoclonal antibodies. Compared to other SARS patients (n = 47), the 3 patients with TB had lower mean CD4+ T cells (368.4/μL vs. 656.6/μL, respectively; p = 0.05) and lower mean CD8+ T cells (371.0/μL vs. 490.1/μL, respectively; p = 0.39). SARS-CoV immunoglobulin G (IgG) antibody titers were measured by enzyme-linked immunosorbent assay kit (Huada Company, Beijing, China) at months 1, 2, 3, 4, 7, 10, and 16 after disease onset. (Titers were not measured for the 3 TB patients at month l.) Compared to most (26 [78.8%] of 33) other SARS patients whose antibodies remained detectable throughout follow-up, 2 of the 3 TB patients (patients 1 and 3) had undetectable antibody titers as of months 7 and 16, respectively. In patient 1, antibody titers, when detectable, were unusually low (40). Both patients 1 and 3 had prolonged viral excretion in stools, sputum, or both. While the median (range) duration of virus excretion in stools and sputa for the entire measurable cohort (n = 56) was 27 (16–127) and 21 (14–52) days, respectively (*3*), it was 125 and 16 days for patient 1, and 109 and 52 days for patient 3 (viral excretion data could not be obtained from patient 2 because sequential specimens for detection were unavailable).

TB in SARS patients has been reported on rare occasions (*4**,**5*). In a cohort of 236 patients in Singapore, it was diagnosed in 2 patients after recovery from SARS (*4*). As with patient 1 in this study, TB had developed after the patient acquired SARS, most likely as the result of reactivation of past infection or new infection with *M. tuberculosis*, while temporarily immunosuppressed because of SARS (*6*) and corticoid therapy. Such phenomena have been described with other viral infections such as measles and HIV (*7**,**8*). By contrast, patients 2 and 3 were known TB patients who acquired SARS through exposure to SARS patients in the same hospital wards. Both diseases are known to be transiently immunosuppressive (*6**,**9*), and their combined effect resulted in more pronounced CD4+ cell decreases in coinfected SARS patients than others. Such immunosuppression also resulted in poorer IgG antibody response in coinfected SARS patients than in others and delayed viral clearance, as shown by longer viral excretion in sputum and stools. While viral excretion could be prolonged in coinfected patients, no virus could be isolated from any RT-PCR–positive specimen collected after 6 weeks of illness, which suggests that excreted viruses were no longer infectious (*3*).

These case reports remind us of the importance of strict isolation of SARS patients, careful use of steroids for their case management, and the possibility of coinfection with TB in SARS patients with incomplete recovery.
